# Greater Neural Adaptations following High- vs. Low-Load Resistance Training

**DOI:** 10.3389/fphys.2017.00331

**Published:** 2017-05-29

**Authors:** Nathaniel D. M. Jenkins, Amelia A. Miramonti, Ethan C. Hill, Cory M. Smith, Kristen C. Cochrane-Snyman, Terry J. Housh, Joel T. Cramer

**Affiliations:** ^1^Applied Neuromuscular Physiology Laboratory, Oklahoma State UniversityStillwater, OK, United States; ^2^Department of Nutrition and Health Sciences, University of Nebraska-LincolnLincoln, NE, United States; ^3^Department of Kinesiology and Health Promotion, California State Polytechnic University, PomonaPomona, CA, United States

**Keywords:** training load, neural adaptations, morphological adaptations, muscle activation

## Abstract

We examined the neuromuscular adaptations following 3 and 6 weeks of 80 vs. 30% one repetition maximum (1RM) resistance training to failure in the leg extensors. Twenty-six men (age = 23.1 ± 4.7 years) were randomly assigned to a high- (80% 1RM; *n* = 13) or low-load (30% 1RM; *n* = 13) resistance training group and completed leg extension resistance training to failure 3 times per week for 6 weeks. Testing was completed at baseline, 3, and 6 weeks of training. During each testing session, ultrasound muscle thickness and echo intensity, 1RM strength, maximal voluntary isometric contraction (MVIC) strength, and contractile properties of the quadriceps femoris were measured. Percent voluntary activation (VA) and electromyographic (EMG) amplitude were measured during MVIC, and during randomly ordered isometric step muscle actions at 10–100% of baseline MVIC. There were similar increases in muscle thickness from Baseline to Week 3 and 6 in the 80 and 30% 1RM groups. However, both 1RM and MVIC strength increased from Baseline to Week 3 and 6 to a greater degree in the 80% than 30% 1RM group. VA during MVIC was also greater in the 80 vs. 30% 1RM group at Week 6, and only training at 80% 1RM elicited a significant increase in EMG amplitude during MVIC. The peak twitch torque to MVIC ratio was also significantly reduced in the 80%, but not 30% 1RM group, at Week 3 and 6. Finally, VA and EMG amplitude were reduced during submaximal torque production as a result of training at 80% 1RM, but not 30% 1RM. Despite eliciting similar hypertrophy, 80% 1RM improved muscle strength more than 30% 1RM, and was accompanied by increases in VA and EMG amplitude during maximal force production. Furthermore, training at 80% 1RM resulted in a decreased neural cost to produce the same relative submaximal torques after training, whereas training at 30% 1RM did not. Therefore, our data suggest that high-load training results in greater neural adaptations that may explain the disparate increases in muscle strength despite similar hypertrophy following high- and low-load training programs.

## Introduction

The neuromuscular system displays a high degree of adaptability and responds to resistance training in a manner that ultimately results in enhanced force or torque production. The specific neuromuscular adaptations responsible for the increase in muscle strength are often broadly grouped as morphological and neural (Folland and Williams, 2007). Several morphological adaptations to resistance training have been identified (Staron et al., 1994; Aagaard et al., 2001; Williamson et al., 2001; Seynnes et al., 2007), the primary and most widely-studied of which is skeletal muscle hypertrophy (Folland and Williams, 2007). It is thought that resistance training also elicits small adaptive changes at multiple sites within the nervous system that, together, enhance muscle strength (Sale, 1988; Gabriel et al., 2006; Lee et al., 2009). One of the primary proposed adaptations is an increase in the ability to maximally excite the motor neuron pool (i.e., agonist activation), which may be secondary to an increase in descending excitatory drive, a decrease in inhibition, and/or an increase in facilitatory mechanisms.

Currently, the recommendations of several leading exercise science and sports medicine organizations is that loads corresponding to 60–85% of one repetition maximum (1RM) should be utilized in order to maximize hypertrophy and strength in response to a resistance training program (NSCA, 2008; Garber et al., 2011). However, several recent experimental studies (Burd et al., 2010; Mitchell et al., 2012; Ogasawara et al., 2013; Jenkins et al., 2016) have called these recommendations in to question. Specifically, low-load (i.e., 30% 1RM) and high-load (i.e., 80–90% 1RM) resistance training have been shown to elicit similar acute increases in muscle protein synthesis (Burd et al., 2010) and comparable muscle hypertrophy in chronic resistance training models (Mitchell et al., 2012; Ogasawara et al., 2013; Jenkins et al., 2016). As a result, there has been a debate (Burd et al., 2013; Schuenke et al., 2013) regarding the most effective resistance exercise loads to prescribe for hypertrophy.

Although, high- and low-load training to failure may elicit similar hypertrophy, high-load training may be superior for enhancing muscle strength (Mitchell et al., 2012; Ogasawara et al., 2013). For example, Mitchell et al. (2012) demonstrated that 80% 1RM leg extension training was superior to 30% 1RM for increasing 1RM, while Ogasawara et al. (2013) demonstrated that 75% 1RM bench press training was superior for increasing maximal voluntary isometric contraction (MVIC) and 1RM strength. Therefore, these data suggest that there may be neural adaptations that facilitate improvements in strength during high-load training that do not occur with low-load training.

Therefore, the purpose of this study was to examine the neuromuscular adaptations, including muscle hypertrophy, muscle activation (i.e., VA and EMG amplitude), and contractile twitch properties, following 3 and 6 weeks of 80 vs. 30% 1RM resistance training to failure in the leg extensors. Based on previous studies (Mitchell et al., 2012; Ogasawara et al., 2013; Jenkins et al., 2016), we hypothesized that resistance training at 80 and 30% 1RM to failure would elicit similar muscle hypertrophy, but that muscle strength would increase to a greater extent following training at 80% 1RM and that this would be accompanied by greater evidence of neural adaptation.

## Methods

### Participants

Thirty men were recruited; however, 4 men did not complete this study. Three men dropped out after enrollment but prior to familiarization and 1 man dropped out during the third week of training due to concerns about the total time commitment. Therefore, only the data from 26 men (mean ± SD; age = 23.1 ± 4.7 years; height = 180.6 ± 6.0 cm; weight = 80.0 ± 14.1 kg) were analyzed and reported in this manuscript. To be eligible, each participant must have been between the ages of 19 and 35, free from any current or ongoing musculoskeletal injuries or neuromuscular disorders involving the hips, knees, or ankles, and could not have completed any regular or formal resistance training for at least 6 months prior to the start of the study. This study was approved by and carried out in accordance with the recommendations of the University of Nebraska-Lincoln's Institutional Review Board for the protection of human participants (IRB Approval #20150715341EP). Prior to any data collection, all participants signed an informed consent form and completed a health history questionnaire.

### Experimental design

A randomized, repeated measures, between-group, parallel design was used for this study. Participants were randomly assigned to either a high- (80% of 1RM; *n* = 13) or low-load (30% of 1RM; *n* = 13) resistance training group, and familiarized with the testing procedures. Participants completed leg extension resistance training to failure 3 times per week for 6 weeks. Testing was completed at baseline, 3, and 6 weeks of training. All participants completed a total of 21 visits, and each visit was separated by 24–96 h and occurred at the same time of day (±2 h). During each testing session, ultrasound, muscle strength, EMG amplitude, VA, and contractile properties were measured. The participants were asked to refrain from any outside resistance exercise for the duration of the study.

### Ultrasound measurements

Muscle thickness and echo intensity were assessed via ultrasound prior to any exercise testing. Transverse ultrasound images of the right leg extensors were obtained using a portable brightness mode (B-mode) ultrasound-imaging device (GE Logiq e, USA) and a multi-frequency linear-array probe (12L-RS; 5–13 MHz; 38.4 mm field-of-view). Images were obtained while the participants were lying in the supine position with their legs extended, relaxed, supported on the table, and their feet braced. Great care was taken to ensure that consistent, minimal pressure was applied with the probe to limit compression of the muscle. To enhance acoustic coupling and reduce near field artifacts, a generous amount of water-soluble transmission gel was applied to the skin. To account for the possibility of non-uniform hypertrophy (Wakahara et al., 2013) all ultrasound measurements were taken at 30, 50, and 70% of the distance from the greater trochanter to the lateral condyle of the femur for the vastus lateralis (VL) (Narici et al., 1989; Wells et al., 2014), at 70, 80, and 90% of the distance between the ASIS and the joint space in front of the anterior border of the medial ligament for the vastus medialis (VM), and at 30, 50, and 70% of the distance from the ASIS to the medial, superior border of the patella for the rectus femoris (RF) (Narici et al., 1989; Korhonen et al., 2009). These locations were marked in permanent ink and kept throughout the duration of the study.

A single, experienced investigator performed all ultrasound scans. The equipment settings for muscle thickness and echo intensity measurements were optimized for image quality using the musculoskeletal mode prior to all testing using a gain of 50 dB, a frequency of 10 MHz, and a depth of 8 cm. These equipment settings were held constant between visits and across participants. All ultrasound image analyses were performed using Image-J Software (National Institutes of Health, USA, version 1.47v). Prior to all analyses, each image was scaled from pixels to cm using the straight-line function in Image-J. The muscle thickness of the leg extensors (i.e., VL, VM, and RF) was measured as the distance (cm) from the adipose tissue-muscle interface to the muscle-bone (for the VM) or muscle-muscle interface (for the VL and RF due to the vastus intermedius lying deep; Radaelli et al., 2013). Muscle thickness was determined using the straight-line function in the Image-J software. Muscle thickness was averaged across the three sites (proximal, middle, and distal) for each muscle (VL, VM, and RF) and then across muscles at Baseline, Week 3, and Week 6 for further analyses.

VL, VM, and RF muscle echo intensity values were assessed by computer-aided gray-scale analysis using the standard histogram function in Image-J and were determined from the maximal rectangular region of interest using the rectangle function that included as much of the muscle of interest as possible without including any surrounding fascia (Caresio et al., 2014). Similar to muscle thickness, echo intensity was averaged across the three sites and three muscles at Baseline, Week 3, and Week 6 for further analyses. The mean echo intensity value was reported as a value between 0 (black) and 255 (white) arbitrary units (au).

### Muscle strength and voluntary activation measurements

For isometric testing, the participants were seated with straps securing the trunk, pelvis, and contralateral thigh on a calibrated isokinetic dynamometer (Biodex System 3; Biodex Medical Systems, Inc. Shirley, NY, USA) with a custom-fitted load cell (Omegadyne, model LC402, range 0–500 lbs, Stamford, CT, USA). The axis of rotation of the dynamometer head was aligned with the lateral epicondyle of the right femur. The seat was tilted back so that there was 120° between the thigh and the trunk to expose the femoral triangle for location of the femoral nerve trunk and delivery of the electrical stimuli. The leg was flexed to 90° between the leg and the horizontal plane, which was used for both voluntary and evoked isometric muscle actions.

The participants completed 2, 3 s warm-up leg extension muscle actions at 50 and 75% of their perceived effort with 30 s of rest between each muscle action. Following the warm-up and 2 min of rest, participants completed 2, 4–5 s MVICs of the leg extensors with 2 min of rest between each muscle action. On each attempt subjects were instructed to contract as “hard and fast” as possible when the investigator said “go.” Loud, verbal encouragement was given during each MVIC.

At baseline, the highest MVIC force recorded was used to calculate the target force levels during the subsequent, randomly-ordered, isometric step muscle actions at 10, 20, 30, 40, 50, 60, 70, 80, and 90% of MVIC. During each step muscle action, the participants were required to trace their force production on an external computer monitor that displayed the real-time digitized force signal overlaid on the target force level. During these trials, doublet stimuli were applied to the femoral nerve in order to assess VA (i.e., interpolated twitch procedure). Three to five s after these submaximal step muscle actions, a doublet stimulus was administered at rest (potentiated twitch). An MVIC was also completed after the 2, 4–5 s MVICs, but prior to the submaximal step muscle actions, during which a doublet stimulus was also applied. Three to five s after this MVIC, a potentiated twitch was evoked. Percent voluntary activation was calculated as (1-[superimposed twitch/potentiated twitch])^*^100 (Allen et al., 1995; Behm et al., 1996) during all submaximal step muscle actions as well as the MVIC. The same absolute forces associated with each randomly ordered percentage of MVIC at baseline were then used during the subsequent testing sessions at week 3 and week 6. If the participant's strength increased at week 3 and/or week 6, they completed the step muscle actions from 10 to 100% MVIC of the absolute force levels established at baseline, in addition to the newly established MVIC.

Transcutaneous electrical stimuli were delivered via a cathode-anode arrangement using a high voltage (maximal voltage = 400 V), constant-current stimulator (Digitimer DS7AH, Herthfordshire, UK). The cathode was a probe placed over the femoral nerve in the lateral most corner of the femoral triangle and the anode was a disposable surface electrode (40 × 50 mm; Digitimer Ltd, Herthfordshire, UK) fixed over the greater trochanter. Optimal stimulation probe location was determined by delivering single low-amperage exploratory stimuli (20–40 mA) with the cathode probe. Probe location was selected based on visual inspections of the twitch force and the compound muscle action potential (M-wave) amplitudes that were displayed on an external computer screen. Once the location was determined, the skin was marked and all further stimuli were delivered at that location. Maximal peak-to-peak M-wave amplitude (M_PP_) and twitch force were achieved by increasing amperage in 20–100 mA increments until a plateau in twitch force and M_PP_ were observed after three consecutive amperage increases. To ensure a supramaximal stimulus, 120% of the stimulus used to evoke the maximal twitch force and M_PP_ was used to evoke the leg extensor muscles with 3 singlet stimuli while the participant was relaxing. Doublet stimuli (200 ms duration square-wave impulse at 100 Hz) were then used to assess voluntary activation. Two men, 1 from the 80% 1RM group and 1 from the 30% 1RM group, were unable to tolerate the doublet stimuli used to determine VA. One of these men (from the 30% 1RM group) was also unable to tolerate the singlet stimuli used to elicit the M-wave and examine contractile properties. Therefore, for VA, a sample size of 12 for each group was used for analyses. For normalized EMG amplitude during voluntary contractions and for the M-wave and contractile properties, a sample size of 13 and 12 were used for the 80 and 30% 1RM groups, respectively.

Following ultrasound and isometric strength testing, 1RM testing was carried out according to the guidelines established by the National Strength and Conditioning Association (NSCA, 2008). Specifically, the participants performed a light warm-up set with 5–10 repetitions at 50% of estimated 1RM, followed by 2–3 heavier warm-up sets of 2–5 repetitions with loads increasing by 10–20% at each set. Participants then began completing trials of 1 repetition with increasing loads (10–20%) until they were no longer able to complete a single repetition. The highest load (kg) successfully lifted through the entire range of motion with proper technique was denoted as the 1RM, which was determined in ≤4 trials for all subjects. Two to four min of rest were allowed between successive warm-up sets and 1RM trials.

### Electromyography

Pre-gelled bipolar surface electrodes (Ag/AgCl, AccuSensor, Lynn Medical, Wixom, MI, USA) were placed on the VL, VM, and RF muscles of the right thigh with an inter-electrode distance of 30 mm. For the VL, the center of the bipolar electrode pair was placed at 66% of the distance between the anterior superior iliac spine (ASIS) and the lateral superior border of the patella (Hermens et al., 1999). The longitudinal axis of the bipolar electrode pair was arranged parallel to the angle of pennation of the VL fibers (~20°; Fukunaga et al., 1997; Lieber and Friden, 2000). For the VM, the center of the bipolar electrode pair was placed at 80% of the distance between the ASIS and the joint space in front of the anterior border of the medial ligament. For the RF, the center of the bipolar electrode pair was placed at 50% of the distance between the ASIS and the medial superior border of the patella. The longitudinal axis of the electrode pair was oriented parallel to the angle of pennation of the VM fibers (~50°; Hermens et al., 1999). A single pre-gelled surface electrode (Ag/AgCl, AccuSensor, Lynn Medical, Wixom, MI, USA) was placed on the lateral condyle of the tibia to serve as the reference electrode. All electrode locations were marked with a permanent marker and were kept throughout the duration of the study. To reduce inter-electrode impedance and increase the signal-to-noise ratio (Beck and Housh, 2008), local areas of the skin were shaved, abraded, and cleaned with isopropyl alcohol prior to the placement of the electrodes. Interelectrode impedance was measured using a digital multimeter (Fluke 179 True RMS Multimeter, Everett, WA, USA) and kept below 2,000 Ω (Beck and Housh, 2008).

### Signal processing

Electromyographic (EMG) and force signals were recorded during all isometric testing. The EMG and force signals were sampled simultaneously at 2 kHz with a Biopac data acquisition system (MP150WSW, Biopac Systems, Inc., Santa Barbara, CA, USA), recorded on a personal computer, and processed off-line with custom written (N.D.M.J.) software (Labview v. 12.0, National Instruments, Austin, TX, USA). The EMG signals were amplified (gain 1,000) using a differential amplifier (EMG 100C, Biopac Systems, Inc., Santa Barbara, CA, USA, bandwidth 1–5,000 Hz) with a common mode rejection ratio of 110 dB min and an impedance of 2M Ω, and zero-meaned. The voluntary and evoked EMG signals were digitally filtered (zero-phase shift 4th-order Butterworth filter) with a band-pass of 10–499 and 10–999 Hz, respectively. The force signals were low-pass filtered (zero-phase shift 4th-order Butterworth filter) with a 15 Hz cutoff. The force obtained from the load cell (N) was multiplied by the lever arm length (m) to calculate torque (Nm). All subsequent analyses were completed on the filtered and scaled signals.

During the evoked muscle actions, peak twitch torque (PTT) was calculated as the highest 5 ms torque value (Nm) obtained after the onset of the evoked twitch. The peak rate of torque development (+*dt/*dt) and rate of relaxation (−*dt/*dt) were calculated as the positive and negative peaks of the first derivate torque signal (Nm s^−1^), respectively. In addition, M_PP_ was calculated as the peak-to-peak amplitude (μV). M-wave duration (M_DUR_) was calculated as the time (ms) from the onset to cessation of the M-wave.

During the MVICs, the torque and EMG amplitude values were calculated from the 500 ms epoch corresponding to the highest average torque value that occurred during the MVIC plateau. During the submaximal isometric step muscle actions, EMG amplitude values were calculated from a 500 ms steady torque epoch that occurred before the delivery of the doublet stimulus (Herda et al., 2008). The EMG amplitude was expressed as the root mean square value in μV during the isometric muscle actions. In order to reduce error due to electrode relocation, subcutaneous fat, and the influence of peripheral factors on the EMG signal (Folland and Williams, 2007; Arabadzhiev et al., 2014), the absolute EMG amplitude values during MVIC and at 10–100% of MVIC at Baseline, Week 3, and Week 6 were normalized to the M_PP_ values at Baseline, Week 3, and Week 6, respectively (Behrens et al., 2015) and was thus considered an indicator of central efferent drive to the quadriceps femoris muscles (Lepers et al., 2001; Trezise et al., 2016). Furthermore, EMG amplitude was average across the VL, VM, and RF to calculate quadriceps femoris muscle activation (EMG_QAMP_) (Trezise et al., 2016). The PTT to MVIC ratio (PTT:MVIC) was also determined by dividing PTT by MVIC at Baseline, Week 3, and Week 6. Because PTT reflects the peripheral properties of skeletal muscle and is, in theory, independent of the influence of descending drive from the CNS, whereas MVIC is a function both of the peripheral properties of skeletal muscle and descending drive, this ratio may provide indirect information regarding peripheral vs. neural adaptations.

### Resistance training

During all 18 training visits, participants completed 3 sets of resistance training to failure with loads corresponding (to the nearest 0.57 kg) to either 80 or 30% of 1RM. Participants were instructed to perform all repetitions through a complete range of motion. A metronome (Pro Metronome, EUMLab, Berlin, Germany) was set to 1 Hz, and participants were instructed to perform the concentric and eccentric phases corresponding with each tick of the metronome so that the concentric and eccentric phases were ~1 s. Verbal instruction and encouragement were provided during each set. Failure was defined as the inability to complete another concentric muscle action through the full range of motion. Two min of rest were provided between sets for both conditions (80 and 30% 1RM). The weight utilized during training was adjusted based on the new 1RM established at the week 3 testing session. The total repetitions performed by each subject were calculated as the sum of the repetitions completed for each set across all sets and exercise sessions. Total time under load was calculated for each subject as the sum of the times to completion for each set across all sets and exercise sessions. Total exercise volume was calculated for each subject as the sum of the product of the number of sets performed, the repetitions completed, and the weight lifted and expressed in total weight (kg) lifted across all exercise sessions.

### Dietary analyses

Participants completed a 3-day dietary log prior to the training period. Participants were instructed to write down all food and drink (except water) consumed on the first 3 days of the training period. These data were entered into an online dietary analysis software (http://www.myfitnesspal.com, MyFitnessPal LLC, San Francisco, CA) that provided calculations of absolute daily energy intake (kcal), as well as protein (g), carbohydrate (g), and fat (g) intakes. The average intakes for energy, protein, carbohydrate, and fat across each 3-day period were recorded.

### Statistical analyses

Six separate independent samples *t*-tests were used to analyze total repetitions, total exercise volume, total time under tension, and average daily energy, carbohydrate, fat, and protein intake between the 80 and 30% 1RM groups. Twelve separate analyses of covariance (ANCOVAs) were used to compare groups (80% 1RM vs. 30% 1RM) for muscle thickness, echo intensity, 1RM strength, MVIC strength, VA during MVIC, EMG_QAMP_ during MVIC, PTT, PTT:MVIC ratio, +*dt/*dt, −*dt/*dt, M_PP_, and M_DUR_ using the respective Baseline scores as the covariate and the Week 3 and/or Week 6 scores as the dependent variable. Multiple a priori planned comparisons were used to analyze the differences within conditions (80% 1RM and 30% 1RM) from Baseline to Week 3 to Week 6 using one-way analyses of variance (ANOVAs) and follow-up Sidak-Bonferroni dependent samples *t*-tests.

Initially, an ANOVA model was used to assess VA and EMG_QAMP_ during the submaximal isometric step muscle actions. For VA and EMG_QAMP_, three-way mixed factorial ANOVAs (time [Baseline vs. Week 3 vs. Week 6] × torque [10% vs. 20% vs. 30% vs. 40% vs. 50% vs. 60% vs. 70% vs. 80% vs. 90% vs. 100% MVIC] × group [80 vs. 30% 1RM]) were used to analyze VA and EMG_QAMP_ during the 10 common isometric torques at each visit. Because these initial analyses revealed time × group interactions, we collapsed the data across torque (for EMG_QAMP_ and VA) and applied an ANCOVA model utilizing the respective Baseline scores as the covariate and the Week 3 or Week 6 scores as the dependent variable. Differences within conditions (80% 1RM and 30% 1RM) from Baseline to Week 3 to Week 6 were then analyzed using one-way analyses of variance (ANOVAs) and follow-up Sidak-Bonferroni corrected dependent samples *t*-tests.

Sphericity was tested for each one way and mixed factorial ANOVA using Mauchly's Test of Sphericity. In cases where the assumption of sphericity was not met, Greenhouse-Geisser corrections (Greenhouse and Geisser, 1959) were applied. Equality of variances were tested using Levene's Test for Equality of Variances for each independent samples *t*-test performed. In cases where the homogeneity of variances assumption was not met, the error term and degrees of freedom were adjusted using the Welch–Satterthwaite method. Partial eta effect sizes ($\eta_{p}^{2}$) were calculated for each ANOVA. Significant main effects were analyzed with Sidak-Bonferroni corrected dependent samples *t*-tests on the marginal means. Cohen's *d* effect sizes (*d*) were calculated for independent samples *t*-tests as described previously (Cohen, 1988). The *d* effect sizes for dependent samples *t*-tests were corrected for dependence among means based on the correlation between means as described by Morris and DeShon (2002). All statistical analyses were completed using IBM SPSS Statistics (v. 22; Armonk, NY) and Microsoft Excel (v. 14.3.2, Redmond, WA) and a type-I error rate was set at 10%.

## Results

### Dietary analyses

Table 1 contains the average daily dietary intake data for the 80 vs. 30% 1RM groups. There were no significant differences in average daily energy intake, or protein, carbohydrate, or fat intake between the 80 and 30% 1RM groups.

**Table 1:** **The means ± standard errors, *p*-values, and Cohen's *d* effect sizes for average daily energy, protein, carbohydrate, and fat intake in the 80 and 30% 1RM groups**.

	**80% 1RM**	**30% 1RM**	***p*-value**	**Cohen's *d***
Energy (kcal)	2242.5 ± 137.4	2248.8 ± 99.5	0.97	−0.01
Protein (g)	97.4 ± 9.8	101.8 ± 7.7	0.76	−0.14
Carbohydrate (g)	289.1 ± 31.9	249.0 ± 17.9	0.35	0.43
Fat (g)	82.9 ± 5.9	92.1 ± 6.1	0.35	−0.42

### Total repetitions, time under load, and exercise volume

Table 2 contains the total repetitions completed, total time under load, and total exercise volume in the 80 vs. 30% 1RM groups. The 80% 1RM group had a lower total time under load and completed fewer repetitions than the 30% 1RM group. However, there was no difference in exercise volume between the 80 and 30% 1RM groups across the 6-week training period.

**Table 2:** **The means ± standard errors, *p*-values, and Cohen's *d* effect sizes for the average repetitions performed, time under tension, and volume performed across all sets and training sessions in the 80 and 30% 1RM groups**.

	**80% 1RM**	**30% 1RM**	***p*-value**	**Cohen's *d***
Total repetitions	558.2 ± 45.1	1751.7 ± 140.8	<0.01	−3.17
Total Time under load (s)	1100.2 ± 66.0	3219.0 ± 200.9	<0.01	−3.93
Total volume (au)	38825.2 ± 3002.7	41170.1 ± 4326.7	0.66	−0.17

*au, arbitrary units*.

### Muscle thickness and echo intensity

#### Muscle thickness

**Figure 1:**
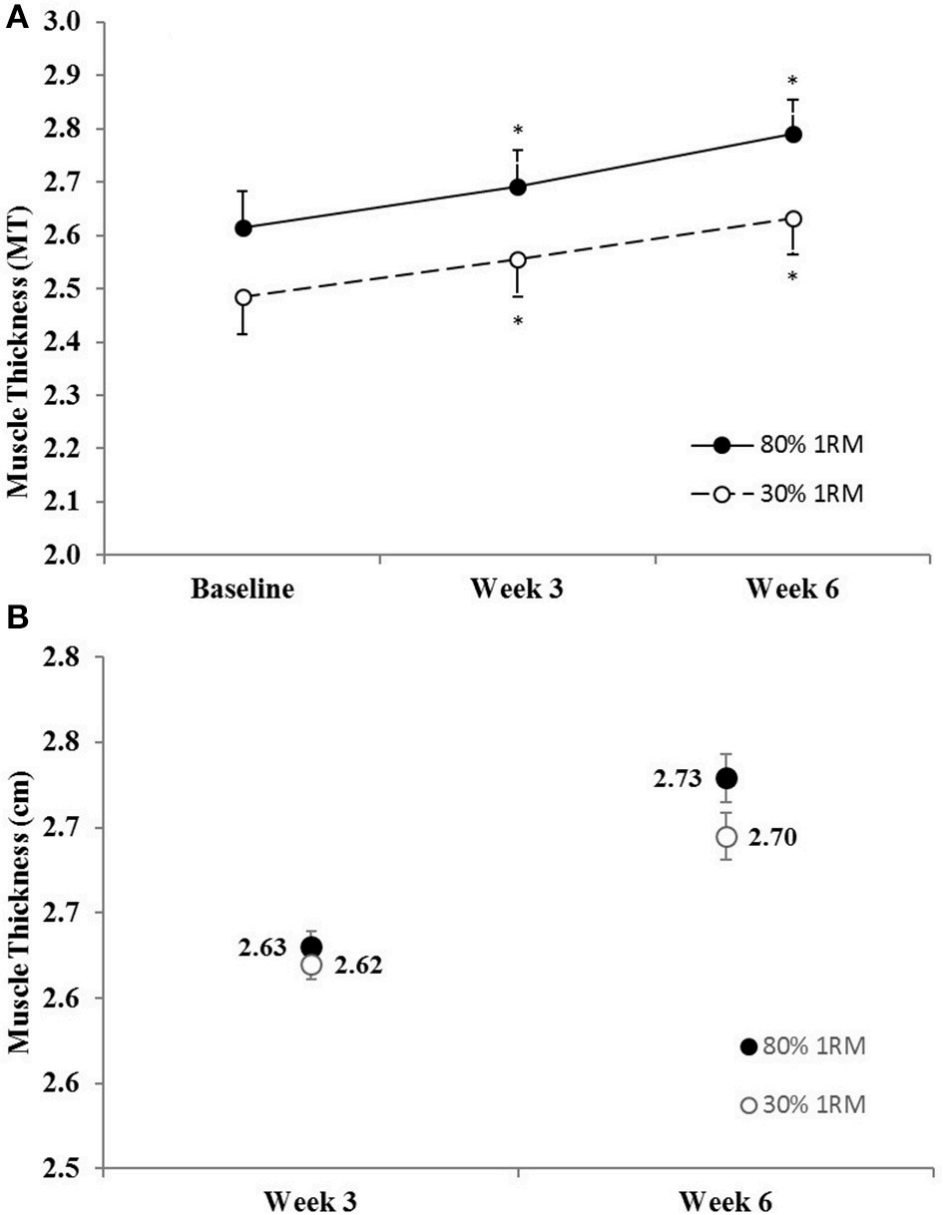
**(A)** Muscle thickness in the 80 and 30% 1RM groups at Baseline, Week 3, and Week 6; and **(B)** adjusted means for muscle thickness in the 80 and 30% 1RM groups at Week 3 and Week 6. Error bars are standard errors. ^*^Indicates a significant increase from Baseline.

There was no difference in the adjusted means for the 80 vs. 30% 1RM groups at Week 3 or Week 6 for muscle thickness (Figure 1B). In the 80% 1RM and 30% 1RM groups, muscle thickness increased from Baseline to Week 3 (*p* < 0.001; *d* = 2.50 and 2.51, respectively), from Week 3 to Week 6 (*p* < 0.001; *d* = 2.94 and 2.92, respectively), and from Baseline to Week 6 (*p* < 0.001; *d* = 3.01 and 4.93, respectively).

#### Echo intensity

**Figure 2:**
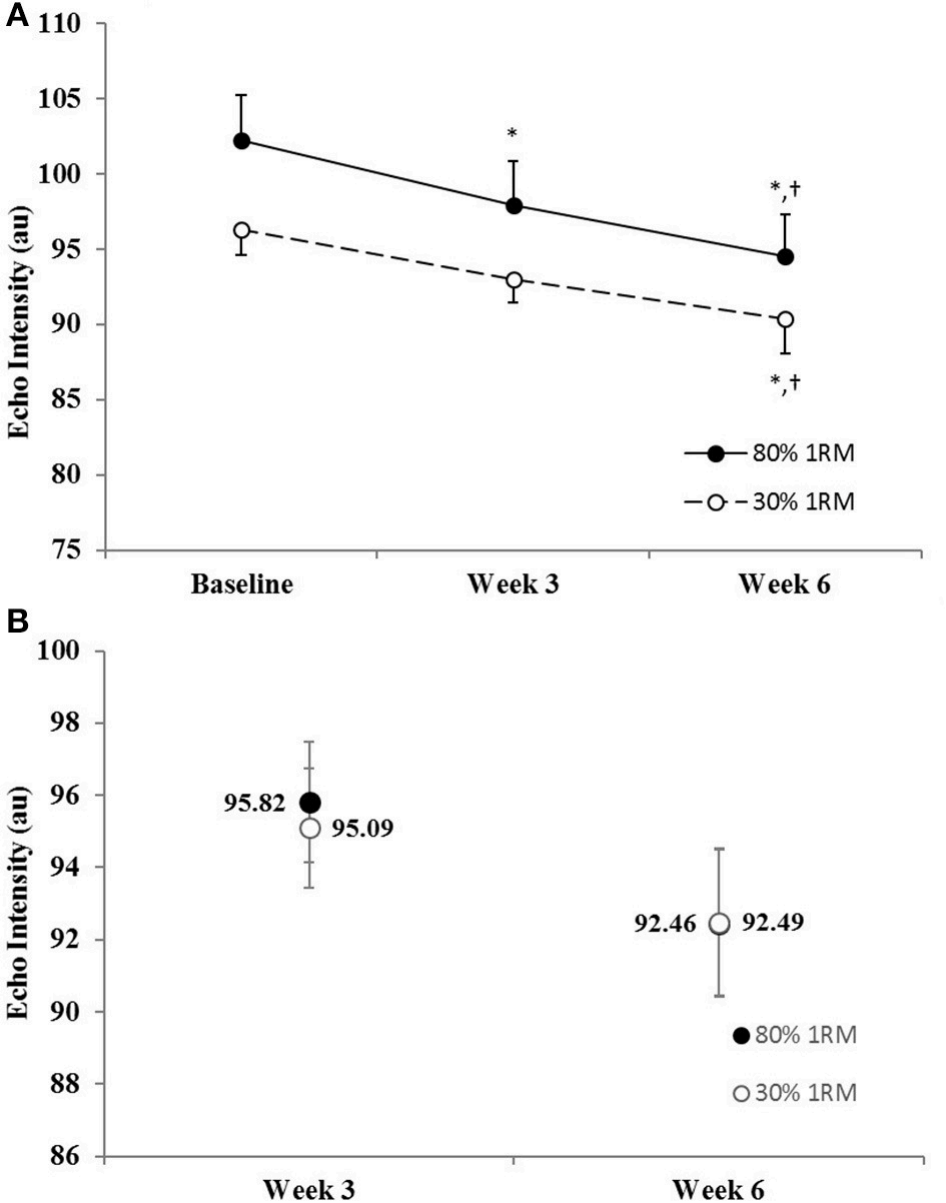
**(A)** Echo intensity in the 80 and 30% 1RM groups at Baseline, Week 3, and Week 6; and **(B)** adjusted means for echo intensity in the 80 and 30% 1RM groups at Week 3 and Week 6. Error bars are standard errors. ^*^Indicates a significant decrease from Baseline. ^†^Indicates a significant decrease from Week 3.

There was no difference in the adjusted means for the 80 vs. 30% 1RM groups at Week 3 or Week 6 for echo intensity (Figure 2B). In the 80% 1RM group, echo intensity decreased from Baseline to Week 3 (*p* = 0.07; *d* = −0.71), from Week 3 to Week 6 (*p* = 0.09; *d* = −0.69), and from Baseline to Week 6 (*p* = 0.004; *d* = −1.17). In the 30% 1RM group, echo intensity did not change from Baseline to Week 3 (*p* = 0.26; *d* = −0.51), but decreased from Week 3 to Week 6 (*p* = 0.07; *d* = −1.22), and from Baseline to Week 6 (*p* = 0.07; *d* = −0.74).

### Muscle strength

#### One repetition maximum

**Figure 3:**
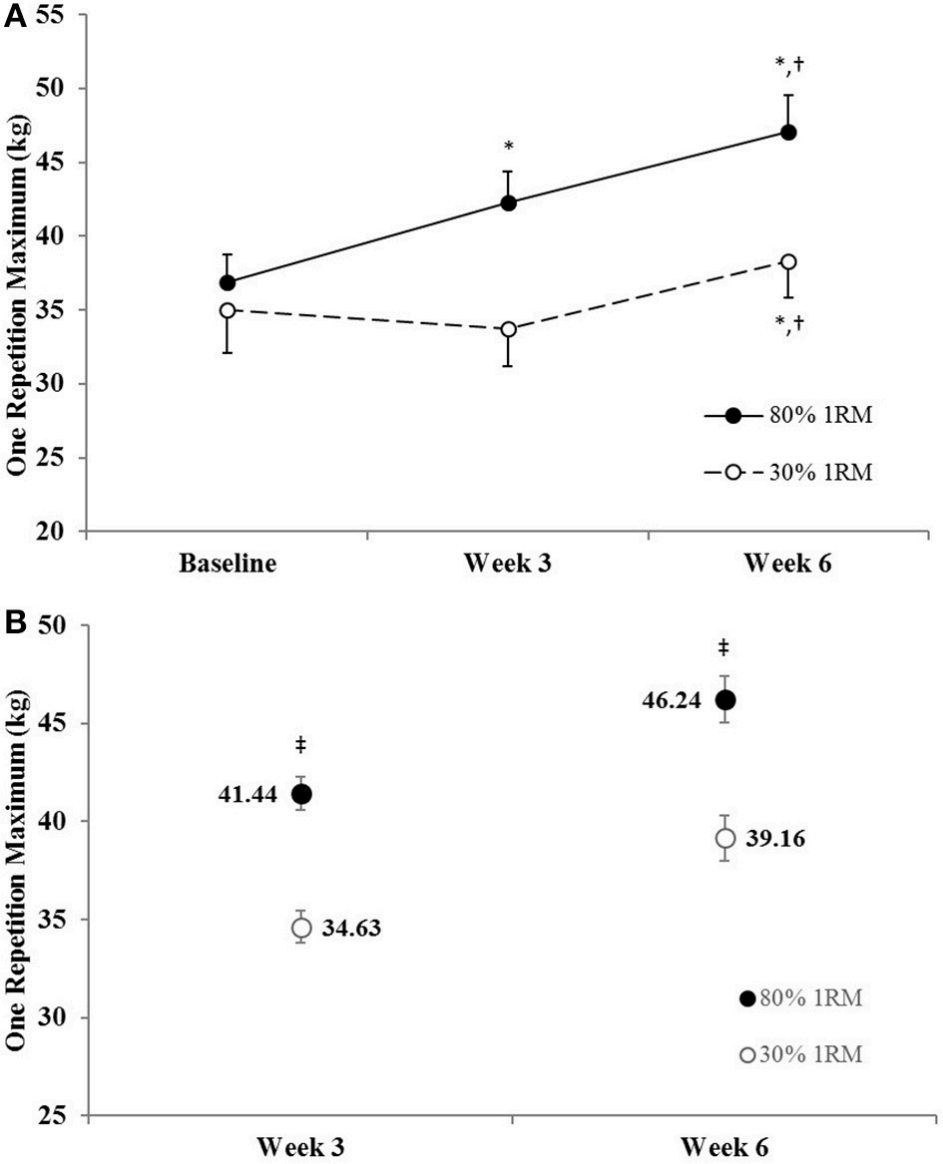
**(A)** One repetition maximum strength in the 80 and 30% 1RM groups at Baseline, Week 3, and Week 6; and **(B)** adjusted means for one repetition maximum strength in the 80 and 30% 1RM groups at Week 3 and Week 6. Error bars are standard errors. ^*^Indicates a significant increase from Baseline. ^†^Indicates a significant increase from Week 3. ^‡^Indicates a significant difference between the 80 and 30% 1RM groups. 80% 1RM > 30% 1RM.

The adjusted means for 1RM strength were significantly greater in the 80% than 30% 1RM group at Week 3 and Week 6 (Figure 3B). In the 80% 1RM group, 1RM strength increased from Baseline to Week 3 (*p* < 0.01; *d* = 2.10), from Week 3 to Week 6 (*p* < 0.01; *d* = 2.10), and from Baseline to Week 6 (*p* < 0.01; *d* = 2.53) in the 80% 1RM group. In the 30% 1RM group, 1RM strength did not change from Baseline to Week 3 (*p* = 0.51; *d* = −0.39), but increased from Week 3 to Week 6 (*p* < 0.01; *d* = 1.74) and from Baseline to Week 6 (*p* = 0.03; *d* = 0.94).

#### Maximum voluntary isometric strength

**Figure 4:**
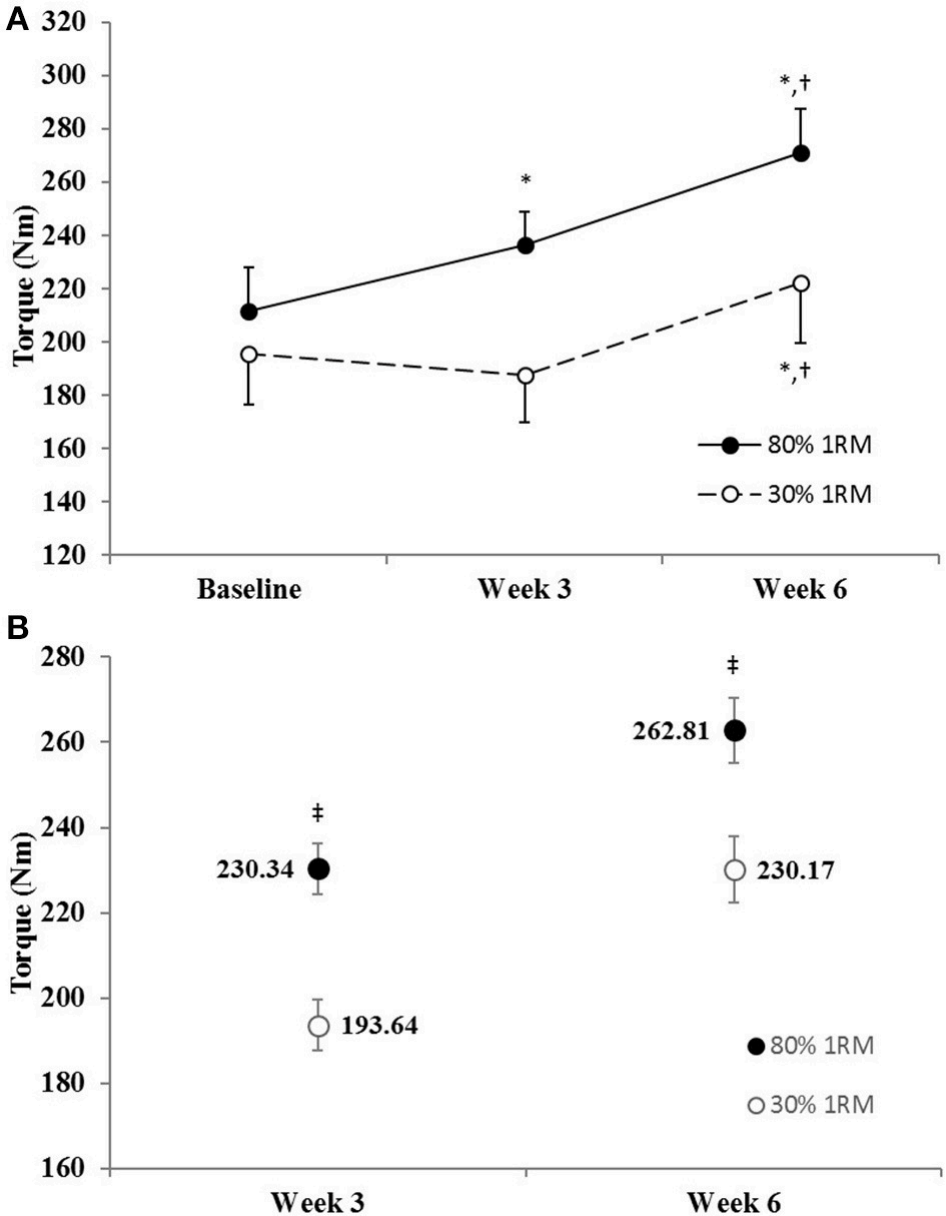
**(A)** Maximum voluntary isometric strength in the 80 and 30% 1RM groups at Baseline, Week 3, and Week 6; and **(B)** adjusted means for maximal voluntary isometric strength in the 80 and 30% 1RM groups at Week 3 and Week 6. Error bars are standard errors. ^*^Indicates a significant increase from Baseline. ^†^Indicates a significant increase from Week 3. ^‡^Indicates a significant difference between the 80 and 30% 1RM groups. 80% 1RM > 30% 1RM.

The adjusted means for MVIC strength were significantly greater in the 80% than 30% 1RM group at Week 3 and Week 6 (Figure 4B). In the 80% 1RM group, MVIC strength increased from Baseline to Week 3 (*p* = 0.02; *d* = 1.08), from Week 3 to Week 6 (*p* < 0.01; *d* = 1.18), and from Baseline to Week 6 (*p* < 0.01; *d* = 2.12). Whereas, in the 30% 1RM group, MVIC strength did not change from Baseline to Week 3 (*p* = 0.51; *d* = −0.38), but increased from Week 3 to Week 6 (*p* < 0.01; *d* = 1.51) and from Baseline to Week 6 (*p* = 0.01; *d* = 1.10).

### Contractile twitch properties

#### Peak twitch torque

The adjusted mean for PTT was significantly greater in the 80% than 30% 1RM group at Week 3, but not at Week 6 (Figure 8B). However, PTT did not change significantly from Baseline to Week 3 or 6 in the 80% 1RM (*p* = 0.22; $\eta_{p}^{2}$ = 0.12) or the 30% 1RM (*p* = 0.59; $\eta_{p}^{2}$ = 0.05) groups (Figure 8A).

#### Peak twitch torque to maximal voluntary strength ratio

There was no difference in the adjusted means for PTT:MVIC in the 80 vs. 30% 1RM groups at Week 3 or Week 6 (**Figure 9B**). In the 80% 1RM group, PTT:MVIC was significantly lower at Week 3 (*p* = 0.02; *d* = −0.93) and Week 6 (*p* = 0.02; *d* = −0.66) than at Baseline; whereas there was no significant change in PTT:MVIC from Baseline to Week 3 or 6 in the 30% 1RM group (*p* = 0.18; $\eta_{p}^{2}$ = 0.15).

#### Twitch peak rate of torque development and peak rate of relaxation

There was no difference in the adjusted means for +*dt*/dt in the 80 vs. 30% 1RM groups at Week 3 or Week 6 (Table 3). In addition, +*dt*/dt did not change significantly from Baseline to Week 3 or 6 in the 80% 1RM (*p* = 0.22; $\eta_{p}^{2}$ = 0.12) or the 30% 1RM (*p* = 0.37; $\eta_{p}^{2}$ = 0.09) groups.

**Table 3:** **The adjusted means ± standard errors for the peak rate of torque development (+*dt*/dt) and peak rate of relaxation (−*dt*/dt) of isometric twitches and the peak-to-peak M-wave amplitude (M_PP_) and duration (M_DUR_) at Weeks 3 and 6 in the 80 and 30% 1RM groups**.

	**Week 3**		**Week 6**	
	**80% 1RM**	**30% 1RM**	**80% 1RM**	**30% 1RM**
+*dt*/dt (Nm s^−1^)	777.08 ± 41.01	671.79 ± 42.82	751.84 ± 46.07	676.26 ± 48.10
−*dt*/dt (Nm s^−1^)	−638.47 ± 30.92^*^	−536.40 ± 32.21	−623.21 ± 48.89	−615.90 ± 50.93
M_PP_ (μV)	7087.42 ± 509.32	7317.79 ± 489.17	7451.50 ± 586.84	6667.14 ± 563.62
M_DUR_ (ms)	30.84 ± 0.91^*^	35.52 ± 0.95	32.96 ± 1.20	35.41 ± 1.25

* *Indicates a significant difference between the 80 and 30% 1RM groups at that time point*.

The adjusted mean for –*dt*/dt was significantly lower in the 80% than 30% 1RM group at Week 3, but not at Week 6. However, −*dt*/dt did not change significantly from Baseline to Week 3 or 6 in the 80% 1RM (*p* = 0.99; $\eta_{p}^{2}$ = < 0.01) or the 30% 1RM (*p* = 0.48; $\eta_{p}^{2}$ = 0.06) groups.

#### M-wave properties

There were no differences in the adjusted means for M_PP_in the 80 vs. 30% 1RM groups at Week 3 or Week 6 (Table 3). M_PP_ also did not change significantly from Baseline to Week 3 or 6 in the 80% 1RM (*p* = 0.74; $\eta_{p}^{2}$ = 0.03) or the 30% 1RM (*p* = 0.50; $\eta_{p}^{2}$ = 0.06) groups.

The adjusted mean for M_DUR_ was significantly greater in the 30% than 80% 1RM group at Week 3, but not at Week 6 (Table 3). M_DUR_ decreased significantly from Baseline to Week 3 (*p* = 0.02; *d* = −1.01), but did not change from Week 3 to Week 6 (*p* = 0.58; *d* = 0.34) or Baseline to Week 6 (*p* = 0.70; *d* = −0.29) in the 80% 1RM group. There was also a significant main effect for time in the 30% 1RM group (*p* = 0.03; $\eta_{p}^{2}$ = 0.27), but follow-up analyses revealed no differences in M_DUR_ at Baseline, Week 3, or Week 6.

### Neuromuscular parameters during maximal voluntary isometric muscle actions

#### Voluntary activation

**Figure 5:**
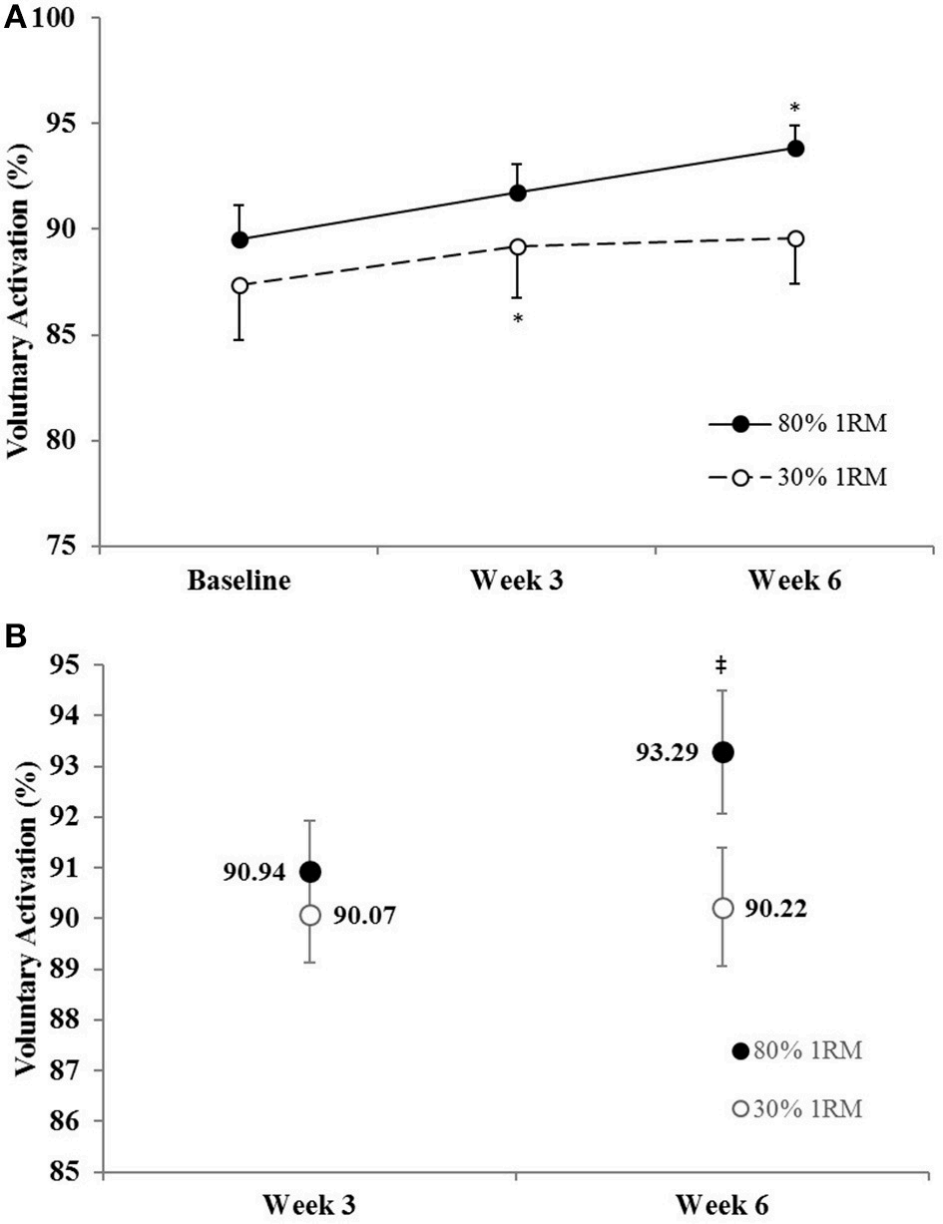
**(A)** Voluntary activation in the 80 and 30% 1RM groups at Baseline, Week 3, and Week 6; and **(B)** adjusted means for voluntary activation in the 80 and 30% 1RM groups at Week 3 and Week 6. Error bars are standard errors. ^*^Indicates a significant increase from Baseline. ^‡^Indicates a significant difference between the 80 and 30% 1RM groups. 80% 1RM > 30% 1RM.

There was no difference in the adjusted means for VA in the 80 vs. 30% 1RM groups at Week 3, but VA was greater in the 80% than 30% 1RM group at Week 6 (Figure 5B). In the 80% 1RM group, VA did not change from Baseline to Week 3 (*p* = 0.29; *d* = 0.49) or Week 3 to Week 6 (*p* = 0.35; *d* = 0.45), but increased from Baseline to Week 6 (*p* = 0.02; *d* = 1.04). In the 30% 1RM group, VA increased from Baseline to Week 3 (*p* = 0.04; *d* = 0.87), but did not change from Week 3 to Week 6 (*p* = 0.99; *d* = 0.07) and was not different from Baseline at Week 6 (*p* = 0.53; *d* = 0.39).

**Figure 6:**
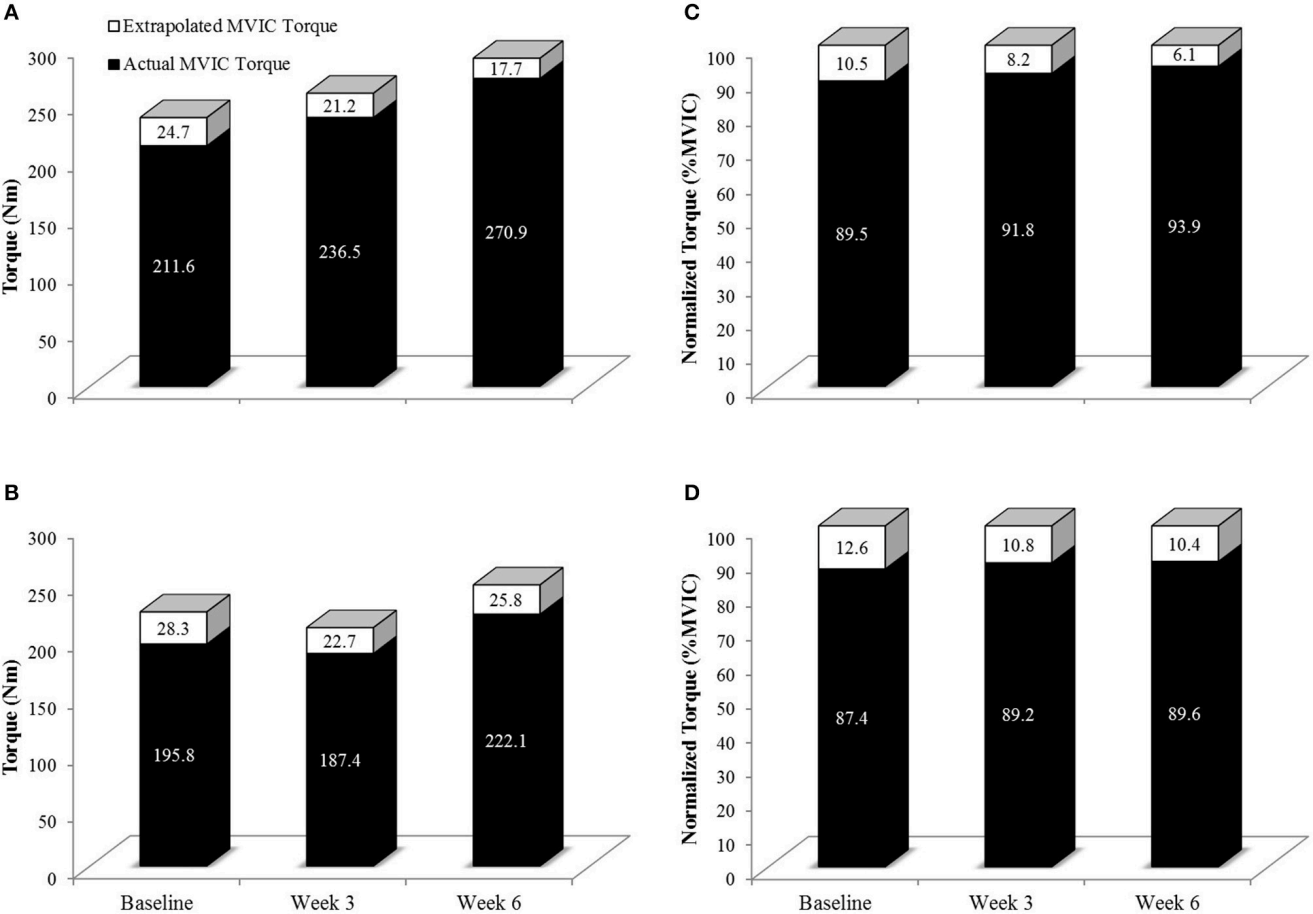
**Actual MVIC torque (Solid Black Bars) and extrapolated MVIC torque (Solid + White Bars) in the 80% 1RM (A,C)** and 30% 1RM **(B,D)** groups at Baseline, Week 3, and Week 6. Extrapolated torque represents the theoretical maximal torque generating capacity of the leg extensors. Note that in **(A,B)**, the y-axis is torque in Nm. In **(C,D)**, torque has been normalized such that the extrapolated torque is equivalent to 100% (thus the units are %MVIC). As can clearly be shown in **(C,D)**, qualitatively, the relative contribution of actual MVIC torque to maximal torque generating capacity increased to a greater degree in the 80% than 30% 1RM groups.

#### Electromyographic amplitude

**Figure 7:**
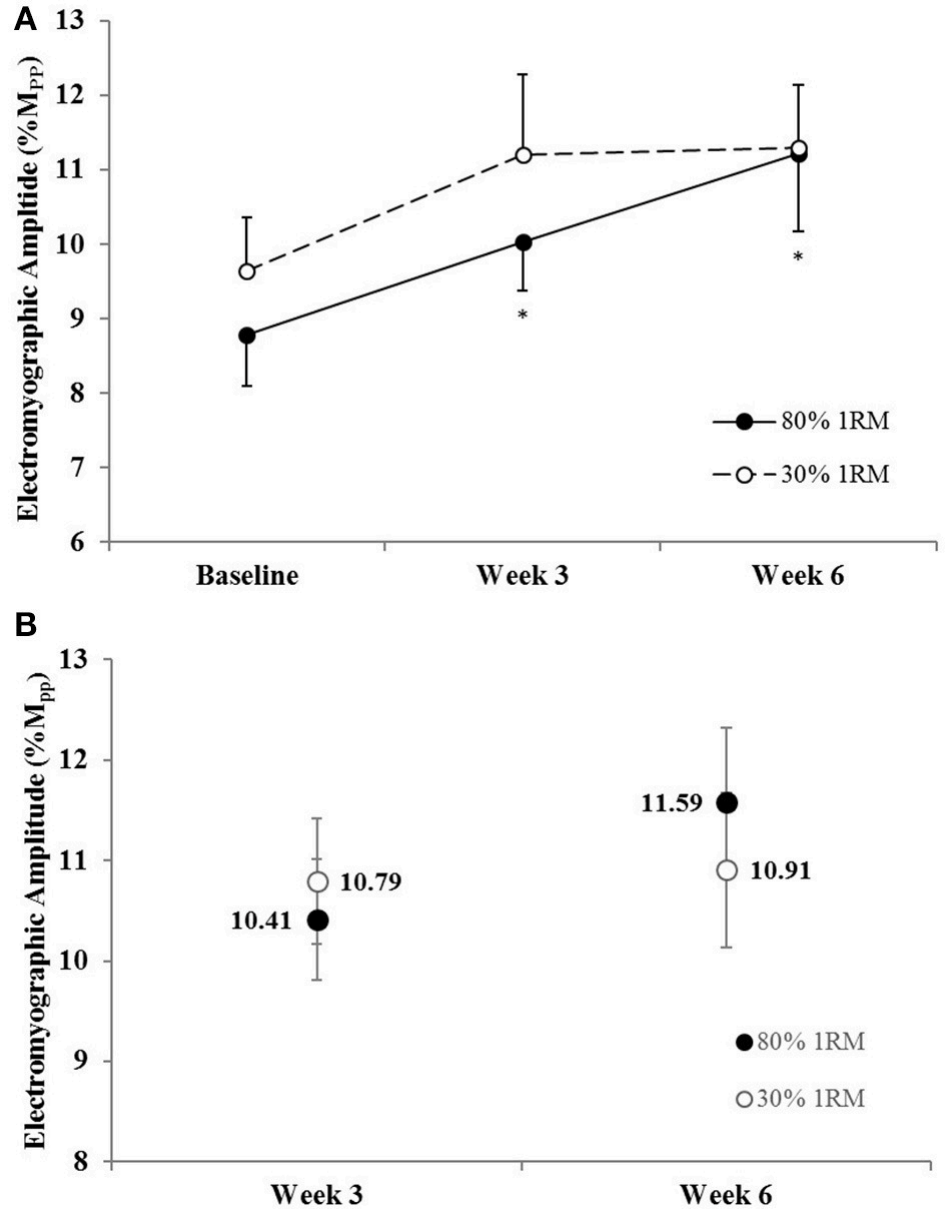
**(A)** Normalized electromyographic amplitude in the 80 and 30% 1RM groups at Baseline, Week 3, and Week 6; and **(B)** adjusted means for electromyographic amplitude in the 80 and 30% 1RM groups at Week 3 and Week 6. Error bars are standard errors. ^*^Indicates a significant increase from Baseline.

There was no difference in the adjusted means for EMG_QAMP_ in the 80 vs. 30% 1RM groups at Week 3 or Week 6 (Figure 7B). In the 80% 1RM group, EMG_QAMP_ increased from Baseline to Week 3 (*p* = 0.05; *d* = 0.76), did not change from Week 3 to Week 6 (*p* = 0.15; *d* = 0.82), and increased from Baseline to Week 6 (*p* = 0.02; *d* = 1.03). In the 30% 1RM group, there was a significant main effect for time (*p* = 0.04; $\eta_{p}^{2}$ = 0.26); however, follow-up analyses revealed no differences in EMG_QAMP_ at Baseline, Week 3, or Week 6.

**Figure 8:**
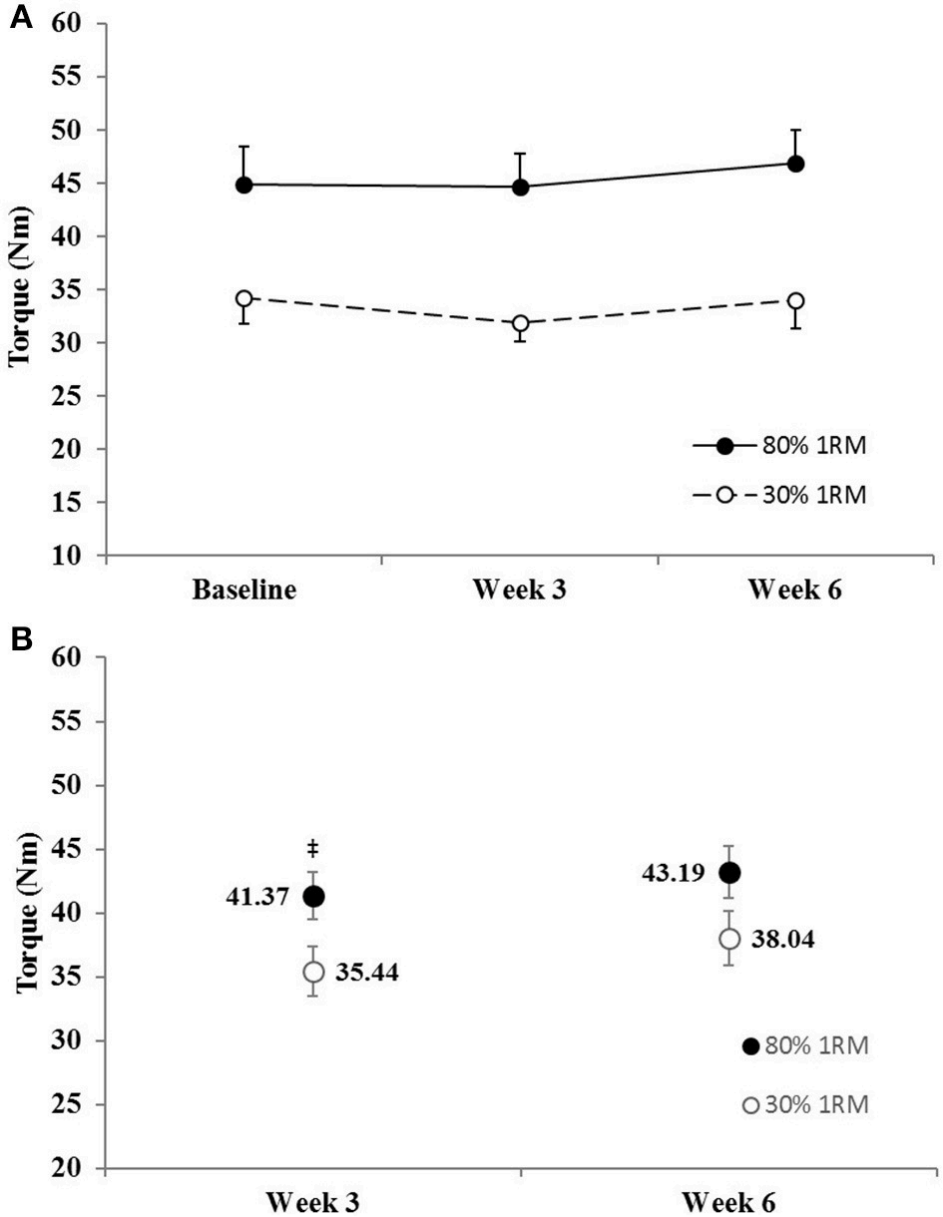
**(A)** Peak twitch torque in the 80 and 30% 1RM groups at Baseline, Week 3, and Week 6; and **(B)** adjusted means for peak twitch torque in the 80 and 30% 1RM groups at Week 3 and Week 6. Error bars are standard errors. ^‡^Indicates a significant difference between the 80 and 30% 1RM groups. 80% 1RM > 30% 1RM.

### Neuromuscular parameters during isometric step muscle actions

#### Voluntary activation

There was no time × torque × group interaction (*p* = 0.69; $\eta_{p}^{2}$ = 0.04) for voluntary activation, but there was a time × group interaction (*p* = 0.03; $\eta_{p}^{2}$ = 0.16) and a main effect for torque (*p* < 0.01; $\eta_{p}^{2}$ = 0.97). Therefore, we collapsed across torque and utilized an ANCOVA model to analyze between group differences at Week 3 and Week 6 and one-way ANOVAs to investigate the change in VA across time within groups (**Figures 10C,D**). The adjusted means for VA during the submaximal isometric step muscle actions were lower in the 80% than 30% 1RM group at Week 3 (52.61 ± 1.70 vs. 59.88 ± 1.70%) and Week 6 (49.43 ± 1.73 vs. 54.71 ± 1.73%). In the 80% 1RM group, voluntary activation did not change from Baseline to Week 3 (*p* = 0.21; *d* = −0.71) or Week 3 to Week 6 (*p* = 0.12; *d* = −0.67), but decreased from Baseline to Week 6 (*p* = 0.02; *d* = −1.17). In the 30% 1RM group, voluntary activation did not change from Baseline to Week 3 (*p* = 0.14; *d* = 0.72), decreased from Week 3 to Week 6 (*p* = 0.02; *d* = −1.02), but did not change from Baseline to Week 6 (*p* = 0.44; *d* = −0.57).

Voluntary activation (collapsed across time and group) increased in a quadratic fashion from 10 to 100% MVIC.

#### Electromyographic amplitude

There was no significant time × torque × group interaction (*p* = 0.19; $\eta_{p}^{2}$ = 0.06), for EMG_QAMP_, but there were time × group (*p* = 0.09; $\eta_{p}^{2}$ = 0.10), time × torque (*p* < 0.001; $\eta_{p}^{2}$ = 0.30), and torque × group (*p* < 0.01; $\eta_{p}^{2}$ = 0.11) interactions (**Figures 11A,B**).

Because we were primarily concerned with the between group changes across time, we further evaluated the time × group interaction by collapsing across torque and utilizing an ANCOVA model to analyze between group differences at Week 3 and Week 6 and one-way ANOVAs to investigate the change in EMG_QAMP_ across time within groups (**Figures 11C,D**). The adjusted mean for EMG_QAMP_ during the submaximal isometric step muscle actions was lower in the 80% than 30% 1RM group at Week 3 (3.33 ± 0.24 vs. 4.30 ± 0.25 %M_pp_), and Week 6 (3.26 ± 0.26 vs. 3.91 ± 0.27 %M_pp_). In the 80% 1RM group, EMG_QAMP_ did not change from Baseline to Week 3 (*p* > 0.10; *d* = −0.65) or Week 3 to Week 6 (*p* = 0.98; *d* = −0.11), but decreased from Baseline to Week 6 (*p* = 0.06; *d* = −0.72). However, there was no change in EMG_QAMP_ from Baseline to Week 3 or 6 in the 30% 1RM group (*p* = 0.29; $\eta_{p}^{2}$ = 0.11).

## Discussion

This was the first study to examine neuromuscular adaptations during maximal and submaximal contractions following 3 and 6 weeks of 80% 1RM vs. 30% 1RM resistance training to failure. The primary results indicated that, despite similar increases in muscle thickness from Baseline to Week 6 for the 80% (6.7%) and 30% 1RM (6.0%) groups (Figure 1), muscle strength increased to a greater degree for the 80% than the 30% 1RM group (Figures 3, 4). Specifically, 1RM and MVIC strength increased from Baseline to Week 6 by 27.7 and 28.0%, respectively, in the 80% 1RM group, whereas 1RM and MVIC strength increased by 9.5 and 13.4% in the 30% 1RM group. These differences in strength were accompanied by evidence of greater neural adaptations during maximal and submaximal torque production in the 80% than 30% 1RM group (Figures 5–7). For example, although training at 30% 1RM elicited an increase in voluntary activation from Baseline to Week 3, the increase in VA from Baseline to Week 6 was only significant in the 80% 1RM group and VA was greater in the 80% than 30% 1RM group at Week 6. Moreover, only training at 80% 1RM elicited a significant increase in EMG_QAMP_ from Baseline to Week 3 and Week 6. Furthermore, we observed decreases in VA and EMG_QAMP_ at submaximal torques in the 80% but not 30% 1RM group (**Figures 10, 11**). Consequently, these data suggest that neural adaptations help explain the greater increases in muscle strength following training with 80% 1RM, despite the similar increases in muscle size following training with 80 and 30% 1RM.

### Morphological adaptations

In the present study, muscle thickness increased by 6.7 and 6.0% in the 80 and 30% 1RM groups, respectively, with no difference between groups (Figure 1). These data add to the growing body of literature that has demonstrated comparable hypertrophic adaptations in response to high- vs. low-load resistance training (Mitchell et al., 2012; Ogasawara et al., 2013; Schoenfeld et al., 2015; Jenkins et al., 2016). Hypertrophy has historically been thought to be minimal during the initial stages of resistance training (Moritani and deVries, 1979; Sale, 1988; Gabriel et al., 2006). Yet, several recent studies (Seynnes et al., 2007; DeFreitas et al., 2011; Jenkins et al., 2016) have shown 4–9% increases in muscle size following 3–4 weeks of resistance training in previously untrained men and women. The present findings supported previous studies (Seynnes et al., 2007; DeFreitas et al., 2011; Mitchell et al., 2012; Ogasawara et al., 2013; Schoenfeld et al., 2015; Jenkins et al., 2016) suggesting that (a) hypertrophy is similar in response to training with high- and low-loads to failure and (b) muscle hypertrophy occurs within the first few weeks of a resistance training program.

Damas et al. (2015) recently proposed that the observed hypertrophy following 3–4 weeks of training may be due, in part, to muscle edema and/or damage from unaccustomed exercise. The authors (Damas et al., 2015) recommended future studies to simultaneously measure muscle damage (i.e., ultrasound echo intensity) to rule out the potential influence of muscular edema on muscle size measurements. Echo intensity would be expected to increase in the presence of muscle damage (Radaelli et al., 2013; Damas et al., 2015). However, in the present study, echo intensity decreased or did not change from Baseline to Week 3 in the 80 and 30% 1RM groups, respectively, and decreased from Baseline to Week 6 in both groups (Figure 2). Therefore, although these factors cannot be completely ruled out, the echo intensity measurements in the present study, as suggested by Damas et al. (2015), indicated that the influence of edema and muscle damage was minimal.

Ultrasound echo intensity has also been used as a surrogate of muscle quality (Pillen et al., 2009; Arts et al., 2012; Fukumoto et al., 2012; Radaelli et al., 2013) and as an indicator of skeletal muscle glycogen content and tissue hydration (Sarvazyan et al., 2005; Hill and Millan, 2014; Nieman et al., 2015). For example, Pillen et al. (2009) demonstrated that increases in interstitial fibrous and fat tissues were associated (*r* = 0.87) with increases in echo intensity. Hill and Millan (2014) and Nieman et al. (2015) showed that changes in rectus femoris and vastus lateralis echo intensity are strongly related (*r* = 0.88–0.92) to changes in skeletal muscle glycogen content following cycling exercise. Although, several previous studies have demonstrated decreases in echo intensity following chronic resistance training (Pinto et al., 2014; Radaelli et al., 2014), Radaelli et al. (2014) suggested that the mechanism for changes in echo intensity is unclear. Resistance training is known to enhance intramuscular glycogen concentrations (MacDougall et al., 1977; Tesch, 1988; NSCA, 2016) and improve cellular hydration in young adults (Ribeiro et al., 2017). When muscle glycogen and water content increase, ultrasound images become hypoechoic, resulting in lower echo intensity values. Thus, it is possible that the observed decreases in ultrasound echo intensity in the present study were due to increased intramuscular glycogen and/or cellular hydration (Sarvazyan et al., 2005; Jenkins, 2016). Incidentally, increased intramuscular water content may increase muscle cross sectional area as measured by MRI (Kristiansen et al., 2014), which is considered the gold standard for assessing skeletal muscle size (Ahtiainen et al., 2010). Although, it is also possible that glycogen and water concentrations influence ultrasound measures of muscle thickness, there are insufficient data in the present study to test this hypothesis. Future studies are needed to examine the impacts of muscle glycogen and intramuscular water on MRI (Kristiansen et al., 2014), peripheral quantitative computed tomography (DeFreitas et al., 2011), and ultrasound-based measurements of muscle size.

### Neural adaptations

**Figure 9:**
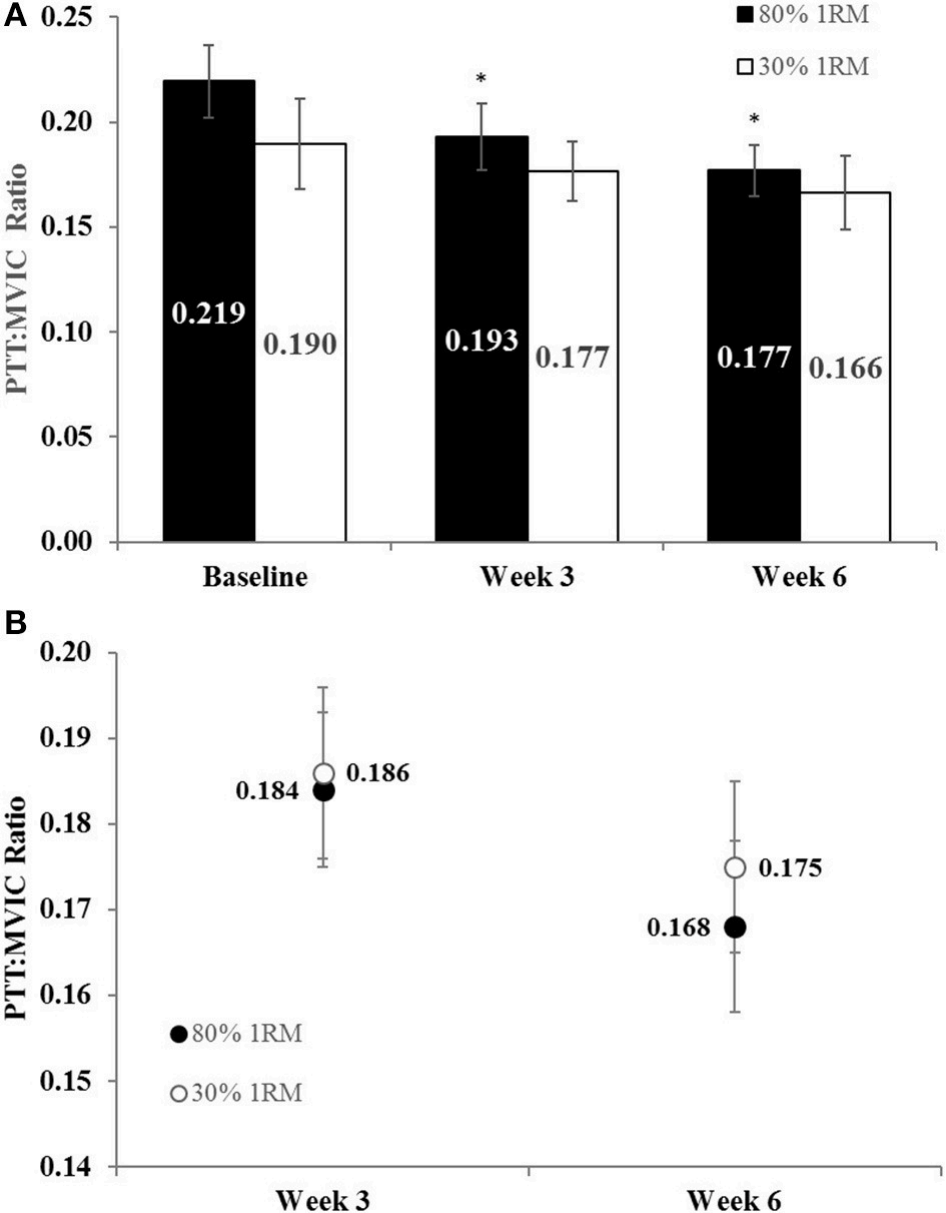
**(A)** The peak twitch torque to maximal voluntary contraction ratio (PTT:MVIC) in the 80 and 30% 1RM groups at Baseline, Week 3, and Week 6; and **(B)** adjusted means for PTT:MVIC in the 80 and 30% 1RM groups at Week 3 and Week 6. Error bars are standard errors. ^*^Indicates a significant decrease from Baseline.

**Figure 10:**
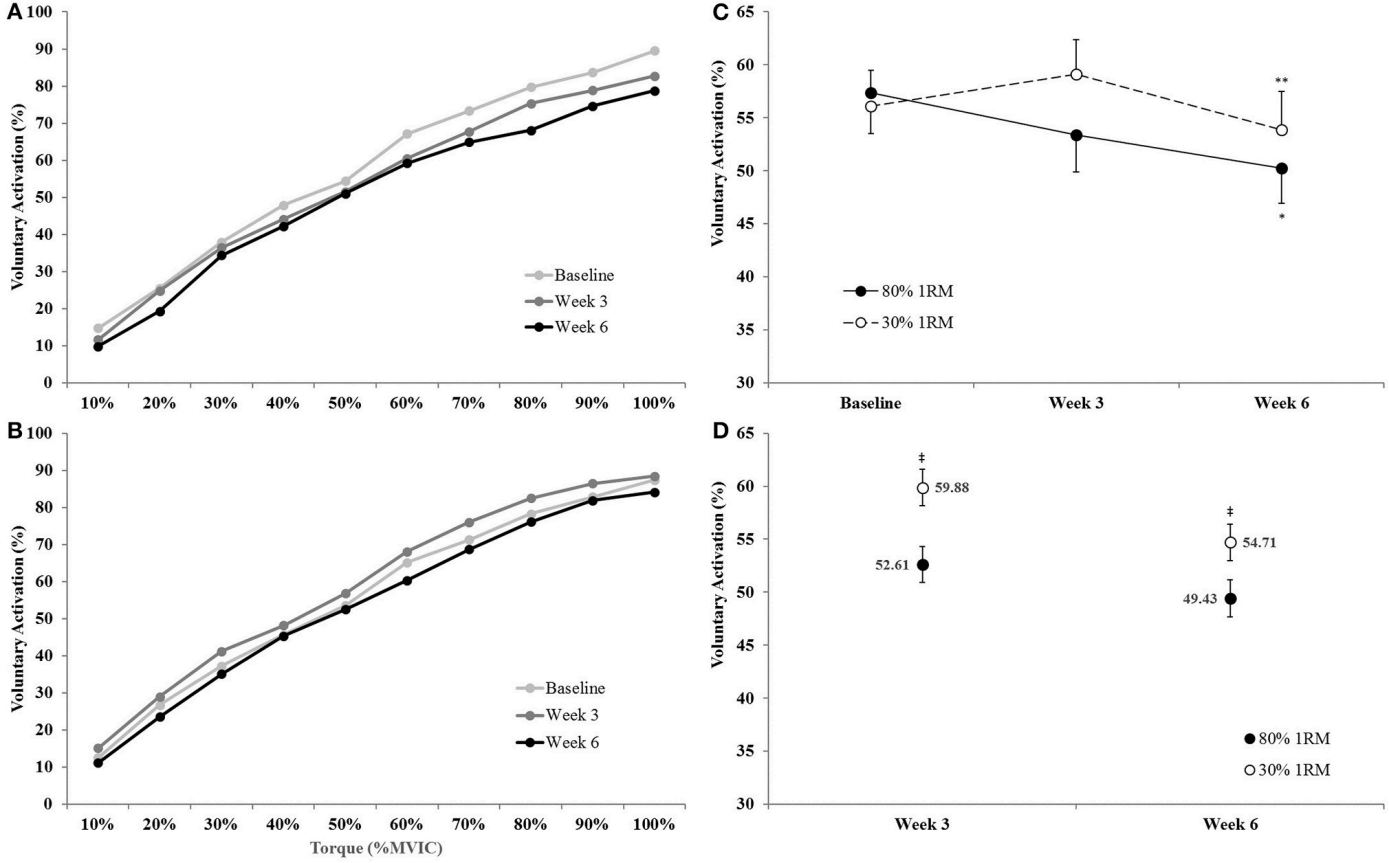
**Voluntary activation from 0 to 100% of the Baseline MVIC at Baseline, Week 3, and Week 6 in the (A)** 80% 1RM and **(B)** 30% 1RM groups; **(C)** voluntary activation (collapsed across torque) in the 80 and 30% 1RM groups; and **(D)** the adjusted means for voluntary activation in the 80 and 30% 1RM groups at Week 3 and Week 6. Error bars are standard errors. ^*^Indicates a significant decrease from Baseline to Week 6. ^**^Indicates a significant decrease from Week 3 to Week 6. ^‡^Indicates a significant difference between the 80 and 30% 1RM groups. 80% 1RM < 30% 1RM.

**Figure 11:**
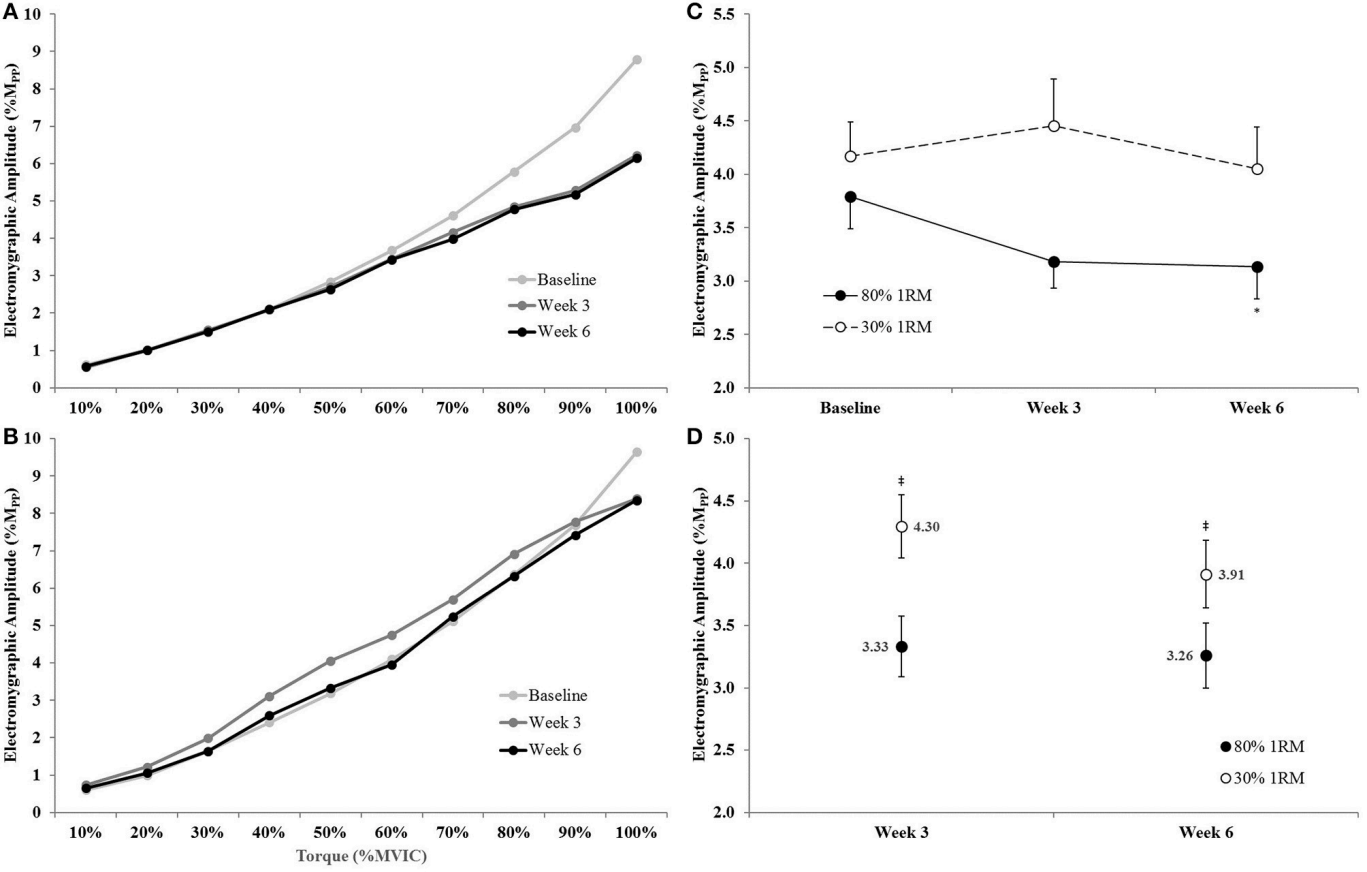
**Normalized electromyographic amplitude (EMG_QAMP_) from 0 to 100% of the Baseline MVIC at Baseline, Week 3, and Week 6 in the (A)** 80% 1RM and **(B)** 30% 1RM groups; **(C)** EMG_QAMP_ (collapsed across torque) in the 80 and 30% 1RM groups; and **(D)** the adjusted means for EMG_QAMP_ in the 80% and 30% 1RM groups at Week 3 and Week 6. Error bars are standard errors. ^*^Indicates a significant decrease from Baseline to Week 6. ^‡^Indicates a significant difference between the 80 and 30% 1RM groups. 80% 1RM < 30% 1RM.

Maximal isometric (e.g., MVIC) and dynamic (e.g., 1RM) strength increased from Baseline to Week 3 in the 80% 1RM group only and increased from Baseline to Week 6 in both the 80 and 30% 1RM groups (Figures 3A, 4A). However, the increases in strength were greater in the 80% 1RM group (Figures 3B, 4B). These different strength adaptations were simultaneously accompanied by group differences in VA and EMG_QAMP_ adaptations (Figures 5–7, 10, 11, respectively) and only the 80% 1RM group showed significant decreases in the PTT:MVIC ratio (Figure 9). Thus, evidence in the present study suggests that 6 weeks of training at 80% 1RM elicited greater neural adaptations than training at 30% 1RM, which may ultimately explain the greater improvements in muscle strength observed following high-load training.

Voluntary activation during MVIC increased 2.2% from Baseline to Week 3 and 4.3% from Baseline to Week 6 in the 80% 1RM group, and was greater for the 80% than 30% 1RM group at Week 6 (Figure 5). Furthermore, the 1.8% increase in VA from Baseline to Week 3 in the 30% 1RM group was not different from the change observed in the 80% 1RM group at Week 3. It has been described that large increases in synaptic input (e.g., motor neuron excitation) are needed to observe small increments in VA at high torques (Herbert and Gandevia, 1999; Kooistra et al., 2007). Herbert and Gandevia (1999) concluded that changes in VA at forces greater than 90% MVIC must indicate large increases in motoneuronal excitation. To illustrate the functional significance of the observed differences in VA, we applied a formula in Figure 6 that was described by Fowles et al. (2000) and Duchateau (1995). This formula extrapolates the maximal torque generating capacity of a muscle from measures of MVIC and VA. Although, criticized (Kooistra et al., 2007), this equation provides a qualitative method to describe changes in VA. Therefore, both the quantitative and qualitative changes in VA described in the present study suggest that 6 weeks of resistance training at 80% 1RM resulted in greater excitation of agonist motor units than training at 30% 1RM. This difference is may be due to a greater augmentation of neural drive (Aagaard et al., 2002; Gabriel et al., 2006; Lee et al., 2009; Behrens et al., 2015) in the 80% 1RM group.

There were no differences in EMG_QAMP_ during MVIC at Week 3 or Week 6 between the 80 and 30% 1RM groups (Figure 7B). However, only training at 80% 1RM elicited a significant increase in EMG_QAMP_ from Baseline to Week 3 and Week 6. Traditionally, training-induced increases in EMG amplitude have been interpreted as increases in neural drive to the muscle (Komi et al., 1978; Moritani and deVries, 1979; Hakkinen and Komi, 1983; Thepaut-Mathieu et al., 1988). While caution is warranted when interpreting changes in surface EMG amplitude in this way, normalizing to the M-wave helps to control for peripheral adaptations and/or changes in electrode placement that may influence the EMG signal (Folland and Williams, 2007; Arabadzhiev et al., 2014), allowing EMG_QAMP_ to be considered an indirect indicator of efferent drive (Lepers et al., 2001; Trezise et al., 2016). In combination with the increase in VA, the increase in EMG_QAMP_ observed in the present study seem to reflect greater motor unit excitation following training at 80% 1RM.

We also examined VA and EMG_QAMP_ during submaximal torque production at the same absolute levels of torque. There was a 12.3% decrease in VA across the submaximal torques from Baseline to Week 6 for the 80% 1RM group (Figure 10C). These decreases were most apparent at high contraction intensities (i.e., 60–100% MVIC; Figure 10A). In the 30% 1RM group, VA displayed a non-significant 5.4% increase from Baseline to Week 3, followed by a significant 8.8% decrease from Week 3 to Week 6 (Figures 10B,C). Furthermore, VA was lower across all submaximal torques at Weeks 3 and 6 in the 80 vs. 30% 1RM group. A decrease in VA at submaximal torque levels suggests a reduced neural cost (i.e., lower activation required to produce the same absolute torque) following training at 80% 1RM. However, in the 30% 1RM group, the level of excitatory input needed to produce the same torques may have slightly increased at Week 3, but decreased back to Baseline levels by Week 6.

The changes in EMG_QAMP_ across submaximal isometric torque observed in the present study were similar to those observed for VA. There was a significant decrease in EMG_QAMP_ across the isometric step muscle actions from Baseline to Week 6 for the 80% 1RM group (Figure 11C). Visual inspection of the EMG_QAMP_ vs. torque relationships (Figure 11A) suggests decreases in EMG_QAMP_ at high contraction intensities (i.e., 70–100% MVIC). Conversely, EMG_QAMP_ did not change in the 30% 1RM group. EMG_QAMP_ was significantly lower in the 80% than 30% 1RM group at Weeks 3 and 6 across submaximal torque levels. Subsequently, much like VA, the EMG_QAMP_ data suggested a reduced neural cost to produce the same absolute torques following training at 80% 1RM, but not 30% 1RM, especially at high torque levels (i.e., 70–100% MVIC; Figure 11A). Our VA and EMG_QAMP_ data during submaximal torque production may also support the findings of Falvo et al. (2010) regarding enhanced “neural economy” following resistance training.

### Conclusions and implications

Six weeks of high- (80% 1RM) and low-load (30% 1RM) resistance training to failure elicited equivalent hypertrophy as measured by ultrasound. However, training at 80% 1RM induced greater strength gains, which has been demonstrated repeatedly (Campos et al., 2002; Mitchell et al., 2012; Ogasawara et al., 2013; Schoenfeld et al., 2015; Jenkins et al., 2016). The unique contributions of this study were the robust measurements (VA and EMG_QAMP_ during maximal and submaximal torque levels) used to elucidate any potential underlying neural factors. Indeed, greater neural adaptations were observed after resistance training at 80% 1RM compared to the 30% 1RM group. Specifically, our data during maximal torque levels suggests that, after 6 weeks of training, 80% 1RM loads elicit greater increases in neural drive than 30% 1RM loads, while our data during submaximal torque levels suggests that resistance training at 80% 1RM increases the efficiency of muscle activation to a greater extent than 30% 1RM training.

## Author contributions

NJ was a substantial contributor to study concept and design, carried out data acquisition, analysis, and interpretation, and was the primary author. AM, EH, CS, and KC helped carry out data acquisition. TH contributed to study design and manuscript revision. JC was the primary manuscript reviser and a substantial contributor to study concept, study design, and interpretation. All authors approved the final version of this manuscript.

### Conflict of interest statement

The authors declare that the research was conducted in the absence of any commercial or financial relationships that could be construed as a potential conflict of interest.
